# Overexpression of the Maize Sulfite Oxidase Increases Sulfate and GSH Levels and Enhances Drought Tolerance in Transgenic Tobacco

**DOI:** 10.3389/fpls.2018.00298

**Published:** 2018-03-12

**Authors:** Zongliang Xia, Ziwei Xu, Yangyang Wei, Meiping Wang

**Affiliations:** ^1^College of Life Science, Henan Agricultural University, Zhengzhou, China; ^2^Key Laboratory of Wheat and Maize Crop Science, Collaborative Innovation Center of Henan Grain Crops, Zhengzhou, China; ^3^Library of Henan Agricultural University, Zhengzhou, China

**Keywords:** maize, overexpression, sulfite oxidase, glutathione, drought

## Abstract

Sulfite oxidase (SO) plays a pivotal role in sulfite metabolism. In our previous study, sulfite-oxidizing function of the *SO* from *Zea mays* (*ZmSO*) was characterized. To date, the knowledge of *ZmSO’s* involvement in abiotic stress response is scarce. In this study, we aimed to investigate the role of *ZmSO* in drought stress. The transcript levels of *ZmSO* were relatively high in leaves and immature embryos of maize plants, and were up-regulated markedly by PEG-induced water stress. Overexpression of *ZmSO* improved drought tolerance in tobacco. *ZmSO*-overexpressing transgenic plants showed higher sulfate and glutathione (GSH) levels but lower hydrogen peroxide (H_2_O_2_) and malondialdehyde (MDA) contents under drought stress, indicating that *ZmSO* confers drought tolerance by enhancing GSH-dependent antioxidant system that scavenged ROS and reduced membrane injury. In addition, the transgenic plants exhibited more increased stomatal response than the wild-type (WT) to water deficit. Interestingly, application of exogenous GSH effectively alleviated growth inhibition in both WT and transgenic plants under drought conditions. qPCR analysis revealed that the expression of several sulfur metabolism-related genes was significantly elevated in the *ZmSO*-overexpressing lines. Taken together, these results imply that *ZmSO* confers enhanced drought tolerance in transgenic tobacco plants possibly through affecting stomatal regulation, GSH-dependent antioxidant system, and sulfur metabolism-related gene expression. *ZmSO* could be exploited for developing drought-tolerant maize varieties in molecular breeding.

## Introduction

Drought is a key environmental stress factor that impacts growth, development, and yield of field crops (Hu and Xiong, 2014; Singh et al., 2015). It is estimated that 43% of total inter-tilled cropland are arid and semi-arid regions worldwide (Koop and Van Leeuwen, 2017).Thus, water deficit has become a severe threat to crop production (Castroluna et al., 2014). Drought stress often leads to morphological, physiological, biochemical, and gene expression changes of crop plants (Shinozaki and Yamaguchi-Shinozaki, 2007; Joshi and Karan, 2013; Joshi et al., 2016). These responses that result in drought tolerance are associated with numerous genetic loci, of which only few have been functionally characterized (Hu and Xiong, 2014). Recently, it has been shown that sulfur (S) metabolism plays an unexpected, yet important role in drought stress tolerance of plants through balancing S metabolites (Chan et al., 2013; Ahmad et al., 2016). Therefore, it is an alternative way for scientists to identify some key S metabolism-related genes in drought response for developing drought tolerant crops.

As a molybdenum-containing enzyme, sulfite oxidase (SO) participates in sulfite metabolism by catalyzing oxidation of toxic sulfite to sulfate (Kappler and Enemark, 2015; Filiz et al., 2017). Over the past several decades, studies on plant SO have been centered on its biochemical properties in *Arabidopsis* (Eilers et al., 2001; Schrader et al., 2003; Hansch et al., 2006; Byrne et al., 2009). Animal SO possesses a molybdenum center and a heme domain, whereas plant SO has a molybdenum center alone (Eilers et al., 2001; Schrader et al., 2003). Moreover, plant SO exhibits sulfite-dependent oxidizing enzyme activity with ferricyanide or molecular oxygen as electron acceptors (Eilers et al., 2001; Hansch et al., 2006; Byrne et al., 2009). In recent years, increasing studies have focused on physiological roles of SO in higher plants. Studies on SO from *Arabidopsis*, tobacco, and tomato have shown that plant SO detoxifies excess sulfite for maintaining sulfite homeostasis by catalyzing the oxidation of sulfite to sulfate in plants (Brychkova et al., 2007, 2013; Lang et al., 2007; Xia et al., 2012a; Filiz et al., 2017). Interestingly, co-regulation of SO and adenosine 5-phosphosulfate reductase (APR) controls sulfate assimilation pathway and stabilizes S distribution by a sulfate-sulfite cycle in *Arabidopsis* (Randewig et al., 2012).

Although much achievements were made in biological function of plant SO, knowledge of functional characterization of SO from cereal crops is still limited. As a major summer crop, maize (*Zea mays*. L) frequently suffers from drought or water deficit stress, which results in serious yield loss (Lopes et al., 2011; Cooper et al., 2014). Previous studies were mostly focused on genes encoding transporters and assimilatory enzymes responsible for sulfate uptake and metabolism, and evidenced that these functional genes participated in nutrient deficiency and heavy metal stress tolerance in maize (Takahashi et al., 2011; Weckopp and Kopriva, 2014; Huang et al., 2018). Unfortunately, molecular function of maize SO in response to drought is scarce. In our previous study, the SO gene from *Z. mays* (*ZmSO*) was cloned; furthermore, its sulfite-dependent oxidizing activity *in vitro* and sulfite detoxifying function *in planta* were characterized (Xia et al., 2012b). The main objective of this study was to investigate the role of *ZmSO* in drought stress response. To this end, we employed *ZmSO*-overexpressing (OE) tobacco transgenic lines to examine their responses to drought stress by physiological, biochemical, metabolite, and gene transcripts analyses. Ultimately, we provide genetic evidence that *ZmSO* confers enhanced drought tolerance in transgenic plants possibly through affecting stomatal regulation, GSH-dependent antioxidant system, and S metabolism-related gene expression.

## Materials and Methods

### Plant Material and Treatments

The maize inbred line Zheng 58 was used in this study. Seeds were germinated in the dark and then transferred to pots with a mixture of vermiculite and soddy soil (1:1) for culture in a growth room as described by us (Huang et al., 2018). Two-week-old seedlings were treated by irrigating 100 mL distilled-water (Control) or 15% (w/v) of PEG8000 (PEG) into soil in each pot to examine response of *ZmSO* to drought stress. Various organs [roots, stems, leaves, tassels, and immature ears (developing ears at 14 days after pollination)] were sampled from 60-day-old maize plants to assay expression profiles of *ZmSO* in adult maize. All the samples were collected at the indicated time points, frozen in liquid N_2_, and stored at -85°C for RNA extraction and qPCR analysis.

### Drought Tolerance Analysis of *ZmSO*-Overexpressing Tobacco Plants

Wild-type (WT) and *ZmSO*-overexpressing tobacco (*Nicotiana tabacum* cv. Xanthi) plants were used to analyze the stress tolerance. These transgenic lines (OE-3 and OE-7) harboring the recombinant construct 35S:*ZmSO* were produced by us previously (Xia et al., 2012b). The sterilized tobacco seeds were germinated on plates containing 1/2 Murashige and Skoog (MS) medium in a growth chamber (16 h light/8 h dark cycle at 23°C). After 7 days, the seedlings of OE-3, OE-7, and WT were transplanted to pots with a mixture of vermiculite and soddy soil (1:1) (4 seedlings per pot and 3–5 pots for each line) to obtain full-growth plants. These tobacco seedlings were cultivated in the growth room, as described by Huo et al. (2016). After additional 3 weeks, these plants were exposed to progressive drought by withholding water until a severe effect of drought (about 2 weeks) was observed. At the moment, soil moisture content was progressively reduced to around 20%. The soil moisture was measured daily using a Soil Moisture Meter (ECA-SW1, TuoPu Bio Co., China) as described previously (Su et al., 2017). And then, these stressed plants were re-watered. After a 3-day-recovery, fresh weight of each plant and remaining leaf chlorophyll content were measured.

### Determination of Chlorophyll Content

Total leaf chlorophyll content was determined using the method reported by Arnon (1949). Leaf samples (0.5 g) from stressed and control tobacco plants were ground in 80% acetone in the dark and then the homogenate was centrifuged at 8,000 × *g* for 10 min at 4°C. Finally, absorbance of the supernatant was measured at the wavelengths of 645 nm, 663 nm, and 652 nm using a spectrophotometer (Hitachi U2000, Japan).

### Application of Exogenous GSH in Transgenic Plants Upon Mannitol-Induced Osmotic Stress

The sterilized tobacco seeds of WT and both OE lines were germinated on plates containing 1/2 MS medium in a growth chamber. After 10 days, these seedlings were transferred to 1/2 MS medium supplemented with 0 (Control), mannitol (300 mM), and mannitol (300 mM) plus GSH (10 mg/L) for vertical growth. Then, residual chlorophyll content and primary root length were measured after 10 days of the treatment.

### Water Loss Rate and Stomatal Aperture Analyses

For water loss measurement, fresh leaves from WT and OE tobacco plants at the same age were detached and placed on dishes to dry at room temperature. Leaf weight was measured by electronic balance every 30 min for 3 h.

The proportion of open stomata was determined by the number of open stomata to that of stomata counted in epidermal peels from leaves of WT and both OE (OE-3 and OE-7) plants upon 9 days of control and drought stress. The sizes of the stomatal apertures smaller than 0.5 μm were regarded as closure. The widths and lengths of open stomata cco plants under control and the stress conditions were measured using a microscope coupled to a CCD camera (Olympus), and the mean size of stomatal apertures in epidermal peels was calculated by the mean ratio of width to length of at least 20 stomatal apertures according to the method as described by us (Xia et al., 2013).

### Determination of H_2_O_2_ and MDA

H_2_O_2_ content was assayed according to the method as described by Xia et al. (2013). Leaf samples (1.0 g) from stressed and unstressed WT and OE tobacco plants were ground and then centrifuged at 80,000 × *g* for 15 min at 4°C, and the supernatant was let react with NH_4_OH (15%, v/v) and TiCl_4_ (10%, w/v). After a second round of centrifuge, the precipitate was washed and dissolved. The resulting solution was used to measure absorbance at the wavelength of 415 nm. Standard H_2_O_2_ samples were also treated with TiCl_4_ and subjected to the same procedure.

MDA content was determined as described by Draper and Hadley (1990) and Huo et al. (2016). Leaf samples from stressed and unstressed WT and OE tobacco plants were ground in 5% (w/v) trichloroacetic acid (TCA) and reacted in 0.67% (w/v) thiobarbituric acid (TBA) for 0.5 h. After cooling and centrifuge, absorbance of the resulting supernatant was measured at the wavelength of 532, 600, and 450 nm, respectively. The MDA content was calculated following the method described by Huo et al. (2016).

### Determination of Sulfate and Glutathione

Sulfate concentration of drought-stressed and unstressed tobacco plants was measured using an ion exchange chromatography system (MIC-1, Metrohm, Switzerland). For sulfate measurement, fresh leaves from stressed and unstressed WT and OE tobacco plants were crushed and dissolved in boiled water. Sulfate was separated and eluted on an IonPac AS9-SC column (4 mm × 250 mm). The eluent solution contained 3.2 mM Na_2_CO_3_ and 1.0 mM NaHCO_3_. The sulfate concentration were determined by the ion chromatography as described previously (Xia et al., 2012b). In the experiment, three replicates were performed for each sample.

Reduced glutathione (GSH) was determined following the method of Griffith (1980). Fresh leaves (0.1 g) from stressed and unstressed WT and OE tobacco plants were homogenized in 1.0 ml of 0.1 M sodium phosphate-EDTA buffer (pH 8.0) and the homogenate was centrifuged at 8,000 × *g* for 10 min at 4°C. And then, absorbance of the supernatant containing phosphate buffer and 5′,5′-dithiobis-2- nitrobenzoic acid (DTNB) was measured at the wavelength of 412 nm.

### Quantitative Real-Time PCR Analysis

Total RNA extraction and the first-strand cDNA synthesis were performed as described previously (Su et al., 2017).The transcript levels of *ZmSO* and several S metabolism-related genes were examined by quantitative real-time PCR (qPCR). The qPCR was performed in 96-well white plates in triplicate on the IQ5 light cycler system (Bio-Rad). The 20-μL reaction mixture consists of 1.0 μL of diluted cDNA, 10 μL of master mixture (SYBR, Thermo Scientific, United States), and 0.5 μM of each gene-specific primer (**Supplementary Table S1**). Relative expression level of each gene was determined according to the 2^-ΔΔCt^ method (Livaka and Schmittgen, 2001). The *Actin2* for tobacco and *Ubiquitin* for maize were used as the reference genes.

### Statistical Analysis

In all the experiments, three biological replicates were performed. Statistical analyses were performed in Excel and SPSS. For all the analyses, the significant level was set at *P* < 0.05 or *P* < 0.01.

## Results

### Transcript Levels of *ZmSO* in Maize Organs and During Water Stress

**FIGURE 1:**
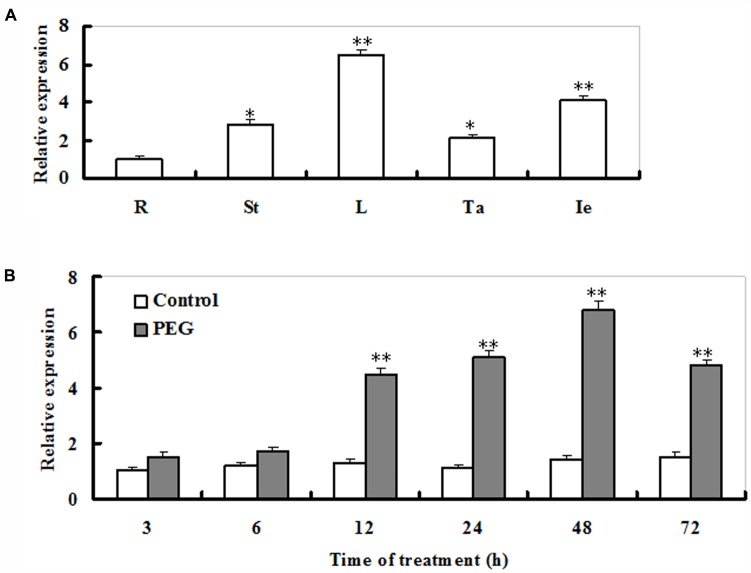
Transcript profiles of *ZmSO* in major organs of maize adult plants and its response to drought stress. **(A)** The transcriptional patterns of *ZmSO* in maize root (R), stem (St), leaf (L), tassel (Ta), and immature ear (Ie) samples evaluated by real-time qPCR. For each qPCR, the transcript levels of maize reference gene *Ubiquitin* were also evaluated in various samples. For each assay, the expression level in roots was defined as 1.0 and three technical replicates were conducted. Data shown are Mean ± SE of three independent experiments. ^∗∗^*t*-test, with *P* < 0.01; ^∗^*t*-test, with *P* < 0.05. **(B)** Time-course analysis of *ZmSO* transcript levels under PEG-induced water stress by real-time qPCR. Two-week-old maize seedlings were exposed to distilled-water (Control) or 15% PEG8000 (PEG) for indicated time points (3, 6, 12, 24, 48, and 72 h), and leaf samples were used for qPCR analysis. For each qPCR, the transcript levels of maize reference gene *Ubiquitin* were also evaluated in various samples. For each experiment, three technical replicates were conducted. Data shown are Mean ± SE of three independent experiments. ^∗∗^*t*-test, with *P* < 0.01.

Transcriptional profiles of *ZmSO* (accession number FJ436404) were examined in roots, stems, leaves, tassels, and immature embryos of adult maize plants. The *ZmSO* transcripts were significantly high in leaves and immature embryos (**Figure 1A**). In contrast, the transcript levels of *ZmSO* were low in roots. Its transcripts in leaves were five times greater than those in roots (**Figure 1A**).

Response of *ZmSO* to PEG-induced water stress in maize seedlings was examined by qPCR. The transcript levels of *ZmSO* displayed a significant increase upon 6 h of PEG exposure, and reached a maximal level at 48 h (more than a 5-fold increase), then gradually decreased and maintained to a relatively high level during 72 h of the treatment (**Figure 1B**).This result showed that the expression of *ZmSO* was up-regulated by water stress.

### Response of *ZmSO*-Overexpressing Tobacco Plants to Drought Stress

**FIGURE 2:**
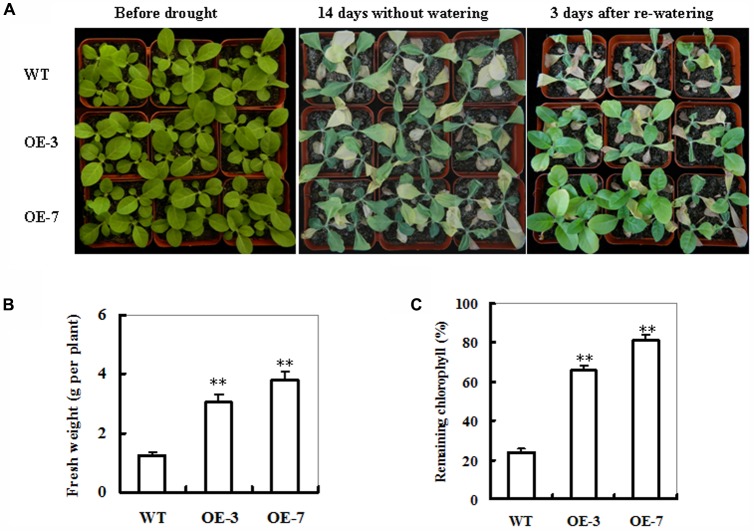
Phenotypes of wild-type (WT) and *ZmSO*-overexpressing tobacco plants in response to drought stress. **(A)** Drought tolerance of potted plants of WT and *ZmSO*-overexpressing (OE) tobacco. Four-week-old WT and transgenic lines (OE-3 and OE-7) were grown in soil in pots for 14 days without watering, and then re-watering for 3 days. **(B)** Fresh weight of 14-day-drought-stressed WT and *ZmSO*-OE plants after 3 days recovery. Values are mean ± SE, *n* = 12. ^∗∗^*t*-test, with *P* < 0.01. **(C)** Relative remaining chlorophyll (%) in 14-day-drought-stressed WT and *ZmSO*-OE plants. Values are mean ± SE, *n* = 12. ^∗∗^*t*-test, with *P* < 0.01.

In our previous study, six homozygous transgenic tobacco lines harboring *35S:ZmSO* expression cassette were constructed (Xia et al., 2012b). Among these lines, both OE-3 and OE-7 had highest SO expression levels in transcripts, protein and activities. Four-week-old *ZmSO*-overexpressing plants were employed to examine their responses to drought stress in soil. As shown in **Figure 2**, under well-watered conditions, there were no obvious differences in leaf size and leaf number between WT and both OE lines (**Figure 2A**; left panel). After 14 days without watering, more than 50% of the WT leaves were turning yellow, soft and even dead, showing severe wilting symptom, whereas most of the OE leaves were still green and fully expanded, displaying signs of moderate water stress (**Figure 2A**; middle panel). Three days after re-watering, only 30% of the WT plants were recovered, while most OE plants (nearly 90%) survived and started growing (**Figure 2A**; right panel). Consistent with their drought-tolerant performance, biomass and remaining chlorophyll content in both OE lines were significantly higher than those in WT plants (170 and 210% increases averagely, respectively) (**Figures 2B,C**). These results demonstrated that overexpression of *ZmSO* improved drought stress tolerance in tobacco plants.

### Changes of Stomatal Apertures in *ZmSO*-Overexpressing Tobacco Plants During Drought Stress

**FIGURE 3:**
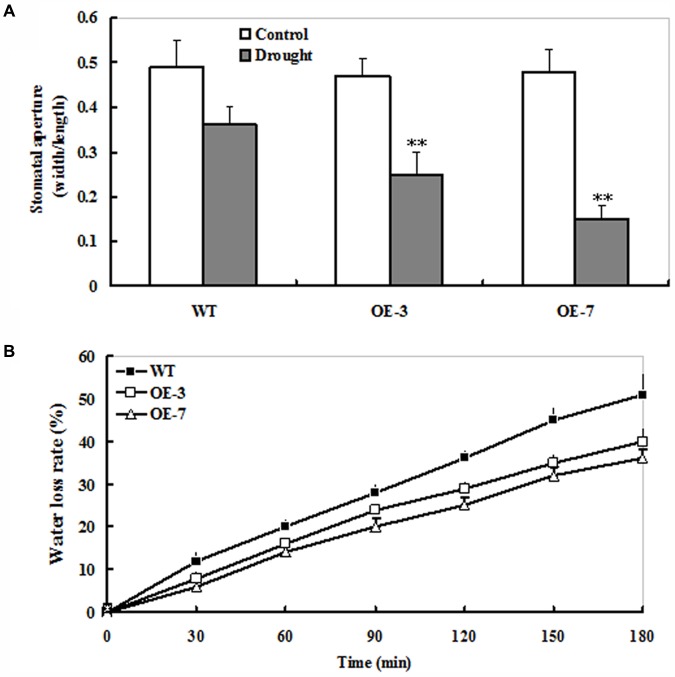
Changes of water loss rate and stomatal apertures in the WT and *ZmSO*-overexpressing tobacco lines under water stress. **(A)** Stomatal apertures. The mean ratio of width to length of at least 20 stomatal apertures was measured in epidermal peels from leaves of WT and both OE (OE-3 and OE-7) plants upon 9 days of drought stress. Bar indicates SE. ^∗∗^*t*-test, with *P* < 0.01. **(B)** Water loss rate. Water loss was monitored by measurement of fresh weight loss from detached leaves of WT and both OE (OE-3 and OE-7) plants at same development age under stressed and control conditions at time intervals indicated. Bars indicate SE, *n* = 6.

The micro-examination of stomatal state was conducted under drought stress. As shown in **Supplementary Figure S1**, the proportion of open stomata was significantly higher in the WT (55%) than those in both transgenic lines (35% for OE-3 and 25% for OE-7, respectively) upon 9 days of drought stress (**Supplementary Figure S1**). Next, leaf stomatal apertures were determined in the WT and OE plants. Upon 9 days of drought stress, stomatal apertures of *ZmSO* transgenic plants were significantly smaller than that of the WT (**Figure 3A**). By contrast, no significant difference in stomatal aperture was observed between WT and OE lines under normal conditions (**Figure 3A**). Furthermore, water loss rate of detached leaves was analyzed from WT and OE plants. During the 3-h period of dehydration treatment, the water loss of detached leaves in both WT and *ZmSO* OE lines displayed markedly linear increases. Noticeably, the WT showed much higher magnitudes of increase in water loss rate than both OE lines (51% for WT and 36% for both OE lines averagely) (**Figure 3B**). These results demonstrated that *ZmSO*-overexpression increased retain water ability and decreased water loss in tobacco plants.

### MDA and H_2_O_2_ Accumulations in *ZmSO*-Overexpressing Tobacco Plants Under Drought Stress

**FIGURE 4:**
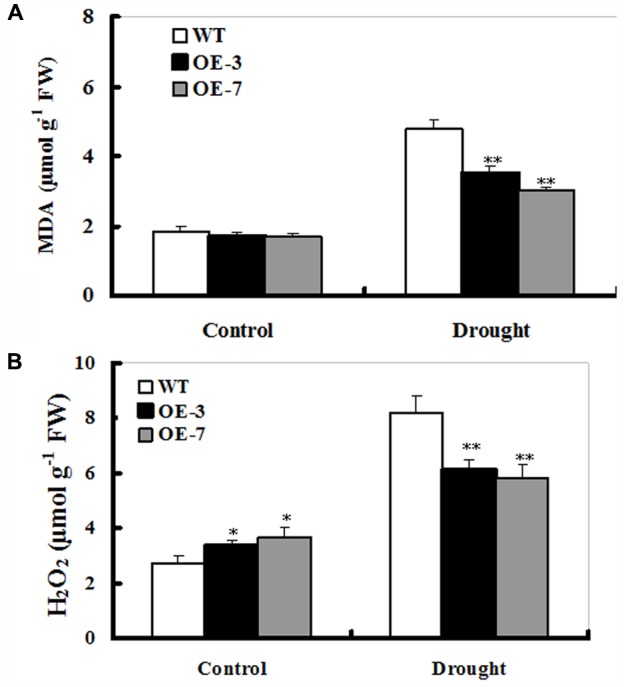
Changes of MDA and H_2_O_2_ in *ZmSO*-overexpressing tobacco lines under drought stress. **(A)** Determination of MDA accumulation in leaves of WT and both OE lines (OE-3 and OE-7) after 9-day drought stress. **(B)** Quantitative determination of H_2_O_2_ accumulation in leaves of WT and both OE lines (OE-3 and OE-7) after 9-day drought stress. In both **(A,B)**, each experiment was repeated three times. Bar indicates SE. ^∗∗^*t*-test, with *P* < 0.01; ^∗^*t*-test, with *P* < 0.05.

Improved drought tolerance conferred by *ZmSO*-overexpression prompted us to detect the difference in lipid peroxidation between WT and OE lines. Malondialdehyde (MDA) was measured between the WT and OE plants after 9 days of drought treatment. The MDA content was significantly higher in the WT (167% increase) than those in both transgenic lines (nearly 90% increase on average), showing that the transgenic plants suffered less membrane damage than the WT (**Figure 4A**).

The low MDA levels in OE plants indicated that these transgenic plants might suffer less oxidative damage than WT during drought stress. Thus, it was of interest to examine reactive oxygen species (ROS) levels in both OE and WT plants under drought conditions. As shown in **Figure 4B**, both WT and OE lines had significant increases in H_2_O_2_ levels upon drought stress. However, both OE lines accumulated lower H_2_O_2_ (only 60% increase averagely) relative to the WT (167% increase) upon drought stress (**Figure 4B**). Additionally, there was a significant difference in H_2_O_2_ levels between WT and both OE lines under unstressed conditions (**Figure 4A**), which might be the cause that SO is a H_2_O_2_ producer in plant cells (Hansch et al., 2006). These results demonstrated that *ZmSO*-overexpressing lines showed less lipid peroxidation and H_2_O_2_ accumulation than the WT under stress conditions.

### Changes in Sulfate and Glutathione (GSH) Levels in *ZmSO*-Overexpressing Tobacco Plants During Drought Stress

**FIGURE 5:**
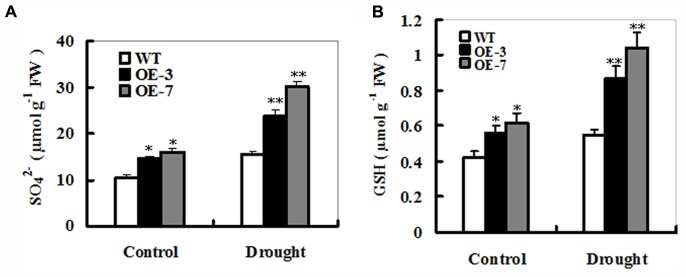
Sulfate and glutathione accumulations in the WT and *ZmSO*-overexpressing tobacco plants under drought stress. Contents of sulfate **(A)** and GSH **(B)** were measured in leaves of WT and both OE lines (OE-3 and OE-7) after 9-day drought stress. Each experiment was repeated three times. Bar indicates SE. Values are mean ± SE. ^∗∗^*t*-test, with *P* < 0.01; ^∗^*t*-test, with *P* < 0.05.

To examine effects of *ZmSO*-overexpression on sulfate and S-metabolites in the sulfate assimilation pathway during drought stress, sulfate and GSH contents were determined under drought and control conditions. As shown in **Figure 5**, drought stress resulted in significant increases in sulfate or GSH levels in both WT and OE lines. For changes in the sulfate levels, both *ZmSO*-transgenic lines showed more increases (64 and 88% increases for OE-3 and OE-7, respectively) than WT plants (only 48% increase) under drought stress (**Figure 5A**). Correspondingly, significant increases in GSH content were detected in both OE lines (68% increase on average), but not in the WT plants (only 30% increase) (**Figure 5B**). Interestingly, under control conditions, total sulfate and GSH levels in both OE lines had significant increases compared to the WT plants (**Figures 5A,B**), showing that increased SO expression resulted in more sulfate and GSH production.

### Growth Inhibition Caused by Drought Stress in Both WT and OE Plants Was Alleviated by GSH

**FIGURE 6:**
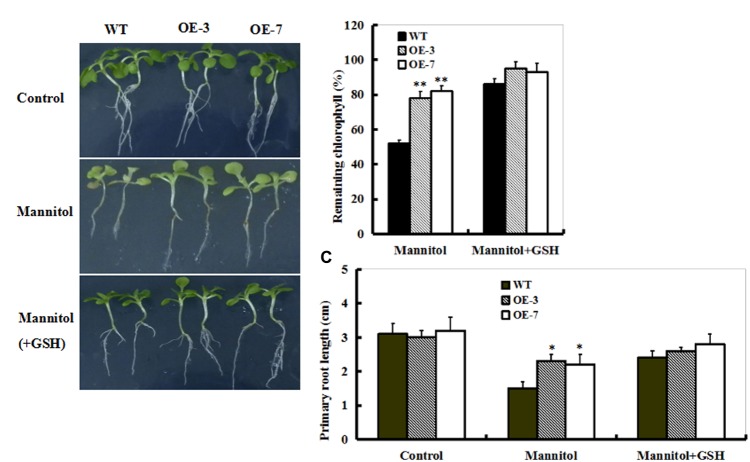
Effect of exogenous GSH on response of WT and *ZmSO*-overexpressing plants to drought stress. **(A)** Representative growth phenotypes of WT and OE tobacco seedlings when exposed to mannitol and mannitol+GSH. Ten-day-old seedlings of WT and OE lines (OE-3 and OE-7) were vertically growing on 1/2 MS medium supplemented with 0 (Control), mannitol (300 mM), and mannitol (300 mM) plus GSH (10 mg/L) for 10 days. **(B)** Relative residual chlorophyll (%) in the WT and OE lines after mannitol and mannitol plus GSH treatments. Values are mean ± SE, *n* = 15. ^∗∗^*t*-test, with *P* < 0.01. **(C)** Primary root length under mannitol treatment in **(B)** after 10 days. Values are mean ± SE, *n* = 15. ^∗^*t*-test, with *P* < 0.01.

To examine this directly, 10-day-old seedlings from WT and OE lines (OE-3 and OE-7) were treated with mannitol or mannitol plus GSH for 10 days. As shown in **Figure 6**, mannitol treatment caused significant reductions in chlorophyll and primary root length, which were alleviated in the presence of GSH in both WT and OE lines (**Figure 6A**). Interestingly, both OE plants exhibited the lowest chlorophyll degradation and root growth inhibition of seedlings among these genotypes of plants (**Figures 6B,C**). This observation indicates that GSH may play a protective role in drought stress.

### Changes in S Metabolism-Related Gene Expression in WT and *ZmSO*-Overexpressing Lines Under Drought Stress

**FIGURE 7:**
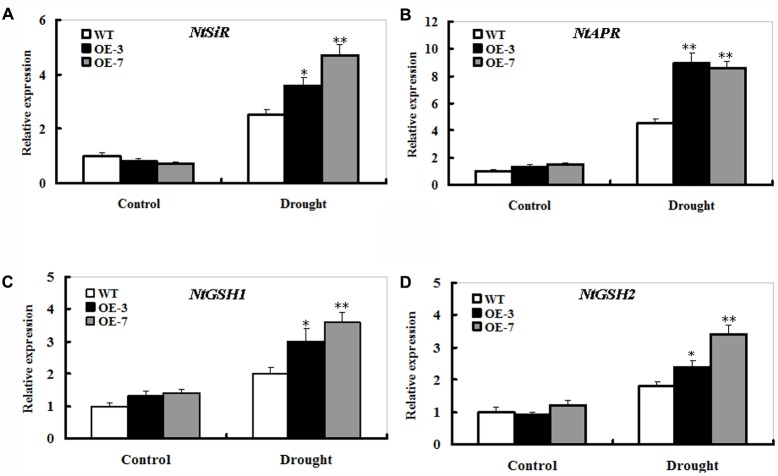
Effect of *ZmSO*-overexpression on transcript levels of the S metabolism-related genes under drought stress. Leaf samples from 9 days of well-watering and drought stress, and transcriptional expression of *NtSiR* **(A)**, *NtAPR* **(B)**, *NtGSH1* **(C)**, and *NtGSH2* **(D)** was detected by qPCR. mRNA levels of these genes were normalized to the transcripts of *Actin2* in the same samples. For each assay, the expression level of WT under control conditions was taken as 1.0, and data represented mean ± SE of three biological replicates. ^∗∗^*t*-test, with *P* < 0.01; ^∗^*t*-test, with *P* < 0.05.

The transcripts of S metabolism-related genes *sulfite reductase* (*SiR), adenosine 5-phosphosulfate reductase* (*APR), γ-glutamylcysteine synthetase* (*GSH1)*, and *glutathione synthetase (GSH2)* were monitored upon drought stress in the WT and *ZmSO*-overexpression plants by qPCR. After 9 days of drought stress, the transcripts of these four genes displayed a trend of significant increase between the WT and OE plants (**Figure 7**). In particular, elevated expression of the *APR* transcripts was quite evident in both OE lines compared with that in the WT (**Figure 7B**). Moreover, the transcripts of *GSH1* in both OE lines were slightly higher than those in the WT even under normal conditions (**Figure 7C**).

## Discussion

Previous studies have shown that SO detoxifies excess sulfite to sulfate, balancing their intracellular ratio (Brychkova et al., 2007; Xia et al., 2012b). Thus, SO plays a regulatory role in the S metabolism pathway in plants. In this study, our genetic evidence suggests that *ZmSO* confers drought tolerance possibly through affecting regulation of stomata opening, GSH-dependent antioxidant system, and S metabolism-related gene expression in tobacco.

### *ZmSO*-Overexpression Increases Sulfate Levels by Sulfite Oxidation During Drought Stress

*ZmSO*-overexpressing plants showed improved drought tolerance when exposed to water deficit (**Figure 2A**). Furthermore, changes in sulfate levels revealed that the greater increase in the sulfate or GSH was observed in both OE lines compared to the WT plants under drought stress (**Figures 5A,B**). In other words, greater amounts of toxic sulfite in the OE plants were transformed to non-toxic sulfate, which in return promoted biosynthesis of the redox buffer GSH. This indicates that SO-dependent sulfite oxidation might have a predominant role in the sulfate metabolism during drought stress. Interestingly, it has been reported that *Hibiscus chlorotic ringspot virus* infection up-regulates plant SO and other S metabolism-related gene transcripts, and increases sulfate and GSH levels for enhanced pathogen defense in kenaf (Zhang and Wong, 2009; Gao et al., 2012). Most recently, Filiz et al. (2017) investigated expression patterns of sulfite scavengers including SO in 10 natural *Arabidopsis* ecotypes and found that transcript levels of SO were up-regulated under heat and high light stresses (Filiz et al., 2017). This result, in combination with our current evidence, further reinforces the view that SO may play an important role in abiotic and biotic stress tolerance by accelerating transformation of sulfite to sulfate, thereby contributing to enhanced biosynthesis of S-containing defense compounds such as GSH in plants. In further work, it is needed to dissect the SO-dependent S metabolism networks during drought stress using multiple-omics analysis in SO-modified plants.

### *ZmSO*-Overexpression Affects Stomatal Aperture During Drought Stress

The stomatal apertures of *ZmSO* OE lines were smaller than those of the WT under drought stress (**Figure 3A**). Moreover, *ZmSO*-overexpression resulted in lower transpiration under water stress (**Figure 3B**). These results suggested that *ZmSO*-overexpression might improve retaining water ability and decrease water loss from plants, likely due to the capability of their stomata to respond promptly to water deficit. As we know, ABA is involved in regulation of stomatal closure during water deficit in plants. Several ABA insensitive mutants such as *abi1* and *abi2* are very susceptible to water deficit because of impaired stomatal aperture regulation (Schroeder et al., 2001). In further studies, it would be interesting to examine whether *ZmSO* is involved in drought stress response in the ABA-dependent pathway.

### *ZmSO*-Overexpression Enhances GSH-Dependent Antioxidant System During Drought Stress

In this study, less MDA and H_2_O_2_ accumulations, which are hallmarks of oxidative stress, were detected in the OE plants upon drought stress (**Figure 4**). Further analysis showed that a significant increase in the GSH content was observed in both OE lines (**Figure 5B**). As we know, GSH is a major organic thiol-containing metabolite, which functions in maintaining redox homeostasis during drought stress (Nagalakshmi and Prasa, 2001; Nahar et al., 2017). Our result demonstrated that amounts of GSH were influenced by the SO levels and in return, GSH levels affected drought stress response of the *ZmSO* OE plants directly (**Figure 6**). The less accumulation of hydrogen peroxide in these OE lines may be a consequence of the higher GSH levels, which strengthened the ROS-scavenging capability. In agreement with our viewpoint, impairment of tobacco glutathione reductase (GR) led to increased sensitivity to oxidative stress because of the reduced GSH regeneration ability (Ding et al., 2009). Thus, it is likely that ZmSO confers drought tolerance by enhancing GSH-dependent antioxidant system that scavenged ROS and reduced membrane injury efficiently.

### The Sulfate Assimilation Pathway Could Affect Adaption of Plants to Environmental Stresses by Regulating Glutathione Metabolism

Plants have the ability to convert inorganic sulfate into reduced sulfur through the reductive sulfate assimilation pathway. In the pathway, several key enzymes such as sulfite reductase (SiR) and adenosine 5-phosphosulfate reductase (APR) can ultimately incorporate sulfate into cysteine, a precursor of glutathione (Leustek and Saito, 1999; Leustek et al., 2000). Glutathione exists with reduced form (γ-Glu-Cys-Gly, GSH) and oxidized form (glutathione disulphide, GSSG) in all living organisms. Glutathione is maintained almost exclusively in the reduced form (GSH) in plants. Thus, it is necessary for plants to maintain a high proportion of GSH (Alscher, 1989). Glutathione is synthesized in the cytoplasm and chloroplast through the catalysis of two enzymes requiring ATP. γ-glutamylcysteine synthetase (GSH1) catalyzes the synthesis of γ-glutamylcysteine, which was shown to be the rate limiting step. Glutathione synthetase (GSH2) adds glycine to γ-glutamylcysteine to produce glutathione (Pasternak et al., 2008). As a major thiol-containing metabolite, GSH is not only an important reduced S sink but a modulator of S assimilation (Hell, 1997). The transcripts of *SiR, APR, GSH1*, and *GSH2* were significantly elevated upon drought stress in *ZmSO*-overexpression plants (**Figure 7**), indicating that the increased levels of sulfate and GSH in the OE plants could be a result of the increased *SO* coupled to enhanced S metabolism-related gene expression during drought stress. Our observations showed that SO conferred drought tolerance in transgenic plants through modulating GSH levels (This study). In support of this viewpoint, tomato plants with impaired SiR significantly decreased GSH levels and showed early leaf senescence (Yarmolinsky et al., 2014). Wang et al. (2016) showed that SiR participated in oxidative stress response possibly by regulating GSH levels in *Arabidopsis* (Wang et al., 2016). Most recently, Lou et al. (2017) have evidenced that applying S nutrition can mitigate Cd toxicity in pakchoi plants by regulating ascorbate-glutathione metabolism (Lou et al., 2017). Similarly, Singh et al. (2017) have demonstrated that S assimilation and its associated metabolites such as cysteine and glutathione play crucial roles in alleviating Cr (VI) toxicity in *Solanum melongena* (Singh et al., 2017). Noticeably, several interesting findings have shown that adequate S provision is important for graminaceous plants to cope with Fe deficiency and Cd toxicity (Astolfi et al., 2012; Celletti et al., 2016).

Taken together, ZmSO can protect plants from drought stress possibly through affecting stomatal apertures, GSH-dependent antioxidant machinery, and S metabolism-related gene expression. In future work, it would be necessary to dissect the mechanism in detail by which the *ZmSO*-dependent sulfite oxidative pathway is involved in drought response in maize. Importantly, *ZmSO* could be exploited for developing drought-tolerant maize varieties by gene transcript-, protein-, or enzymatic activity-assisted selection in molecular breeding program. This might be a new strategy for plant scientists in enhancing drought tolerance of crops.

## Author Contributions

ZXia designed the research. ZXia, ZXu, YW, and MW performed the research and conducted the data analyses. ZXia wrote and revised the manuscript.

## Conflict of Interest Statement

The authors declare that the research was conducted in the absence of any commercial or financial relationships that could be construed as a potential conflict of interest. The reviewer SC and handling Editor declared their shared affiliation.

## Supplementary Material

FIGURE S1 The proportion of open stomata in the wild-type (WT) and *ZmSO*- overexpressing tobacco lines under water stress. The proportion of open stomata was determined by the number of open stomata to that of stomata counted in epidermal peels from leaves of WT and both OE (OE-3 and OE-7) plants upon 9 days of control and drought stress. Bar indicates SE. ^∗∗^*t*-test, with *P* < 0.01.

TABLE S1 qPCR primers used in this study.

## References

[B1] Ahmad N. Malagoli M. Wirtz M. Hell R. (2016). Drought stress in maize causes differential acclimation responses of glutathione and sulfur metabolism in leaves and roots. *BMC Plant Biol.* 16:247. 10.1186/s12870-016-0940-z 27829370 PMC5103438

[B2] Alscher R. G. (1989). Biosynthesis and antioxidant function of glutathione in plants. *Physiol. Plant* 77 457–464. 10.1111/j.1399-3054.1989.tb05667.x

[B3] Arnon D. I. (1949). Copper enzymes in isolated chloroplasts: polyphenoloxidase in *Beta vulgaris*. *Plant Physiol.* 24 1–15. 10.1104/pp.24.1.1 16654194 PMC437905

[B4] Astolfi S. Zuchi S. Neumann G. Cesco S. Sanità di Toppi L. Pinton R. (2012). Response of barley plants to Fe deficiency and Cd contamination as affected by S starvation. *J. Exp. Bot.* 63 1241–1250. 10.1093/jxb/err344 22090437

[B5] Brychkova G. Grishkevich V. Fluhr R. Sagi M. (2013). An essential role for tomato sulfite oxidase and enzymes of the sulfite network in maintaining leaf sulfite homeostasis. *Plant Physiol.* 161 148–164. 10.1104/pp.112.208660 23148079 PMC3532248

[B6] Brychkova G. Xia Z. Yang G. Yesbergenova Z. Zhang Z. Davydov O. (2007). Sulfite oxidase protects plants against sulfur dioxide toxicity. *Plant J.* 50 696–709. 10.1111/j.1365-313X.2007.03080.x 17425719

[B7] Byrne R. S. Hänsch R. Mendel R. R. Hille R. (2009). Oxidative half-reaction of *Arabidopsis thaliana* sulfite oxidase: generation of superoxide by a peroxisomal enzyme. *J. Biol. Chem.* 284 35479–35484. 10.1074/jbc.M109.067355 19875441 PMC2790977

[B8] Castroluna A. Ruiz O. M. Quiroga A. M. (2014). Effects of salinity and drought stress on germination, biomass and growth in three varieties of *Medicagosativa* L. *Avances Invest. Agropec.* 18 39–50.

[B9] Celletti S. Paolacci A. R. Mimmo T. Pii Y. Cesco S. Ciaffi M. (2016). The effect of excess sulfate supply on iron accumulation in three graminaceous plants at the early vegetative phase. *Environ. Exp. Bot.* 128 31–38. 10.1016/j.envexpbot.2016.04.004

[B10] Chan K. X. Wirtz M. Phua S. Y. Estavillo G. M. Pogson B. J. (2013). Balancing metabolites in drought: the sulfur assimilation conundrum. *Trends Plant Sci.* 18 18–29. 10.1016/j.tplants.2012.07.005 23040678

[B11] Cooper M. Gho C. Leafgren R. Tang T. Messina C. (2014). Breeding drought-tolerant maize hybrids for the US corn-belt: discovery to product. *J. Exp. Bot.* 65 6191–6204. 10.1093/jxb/eru064 24596174

[B12] Ding S. Lu Q. Zhang Y. Yang Z. Wen X. Zhang L. (2009). Enhanced sensitivity to oxidative stress in transgenic tobacco plants with decreased glutathione reductase activity leads to a decrease in ascorbate pool and ascorbate redox state. *Plant Mol. Biol.* 69 577–592. 10.1007/s11103-008-9440-3 19043665

[B13] Draper H. H. Hadley M. (1990). Malondialdehyde determination as index of lipid peroxidation. *Methods Enzymol.* 86 421–431. 10.1016/0076-6879(90)86135-I 2233309

[B14] Eilers T. Schwarz G. Brinkmann H. Witt C. Richter T. Nieder J. (2001). Identification and biochemical characterization of *Arabidopsis thaliana* sulfite oxidase. A new player in plant sulfur metabolism. *J. Biol. Chem.* 276 46989–46994. 10.1074/jbc.M108078200 11598126

[B15] Filiz E. Vatansever R. Ozyigit I. I. (2017). Insights into a key sulfite scavenger enzyme sulfite oxidase (SOX) gene in plants. *Physiol. Mol. Biol. Plants* 23 385–395. 10.1007/s12298-017-0433-z 28461726 PMC5391365

[B16] Gao R. Ng F. K. Liu P. Wong S. M. (2012). *Hibiscus chlorotic* ringspot virus coat protein upregulates sulfur metabolism genes for enhanced pathogen defense. *Mol. Plant Microbe Interact.* 25 1574–1583. 10.1094/MPMI-08-12-0203-R 23134059

[B17] Griffith O. W. (1980). Determination of glutathione and glutathione disulfide using glutathione reductase and 2-vinylpyridine. *Anal. Biochem.* 106 207–212. 10.1016/0003-2697(80)90139-6 7416462

[B18] Hansch R. Lang C. Riebeseel E. Lindigkeit R. Gessler A. Rennenberg H. (2006). Plant sulfite oxidase as a novel producer of H2O2–Combination of enzyme catalysis with a subsequent non-enzymatic reaction step. *J. Biol. Chem.* 281 6884–6888. 10.1074/jbc.M513054200 16407262

[B19] Hell R. (1997). Molecular physiology of plant sulfur metabolism. *Planta* 202 138–148. 10.1007/s004250050112 9202491

[B20] Hu H. Xiong L. (2014). Genetic engineering and breeding of drought-resistant crops. *Annu. Rev. Plant Biol.* 65 715–741. 10.1146/annurev-arplant-050213-040000 24313844

[B21] Huang Q. Wang M. Xia Z. (2018). The SULTR gene family in maize (*Zea mays* L.): gene cloning and expression analyses under sulfate starvation and abiotic stress. *J. Plant Physiol.* 220 24–33. 10.1016/j.jplph.2017.10.010 29145069

[B22] Huo Y. Wang M. Wei Y. Xia Z. (2016). Overexpression of the Maize psbA gene enhances drought tolerance through regulating antioxidant system, photosynthetic capability, and stress defense gene expression in tobacco. *Front. Plant Sci.* 6:1223. 10.3389/fpls.2015.01223 26793207 PMC4709446

[B23] Joshi R. Karan R. (2013). “Physiological, biochemical and molecular mechanisms of drought tolerance in plants,” in *Molecular Approaches in Plant Abiotic Stress*, eds Gaur R. K. Sharma P. (Boca Raton, FL: CRC Press), 209–231. 10.1201/b15538-14

[B24] Joshi R. Wani S. H. Singh B. Bohra A. Dar Z. A. Lone A. A. (2016). Transcription factors and plants response to drought stress: current understanding and future directions. *Front. Plant Sci.* 7:1029. 10.3389/fpls.2016.01029 27471513 PMC4943945

[B25] Kappler U. Enemark J. H. (2015). Sulfite-oxidizing enzymes. *J. Biol. Inorg. Chem.* 20 253–264. 10.1007/s00775-014-1197-3 25261289

[B26] Koop S. H. A. Van Leeuwen C. J. (2017). The challenges of water, waste and climate change in cities. *Environ. Dev. Sustain.* 19 385–418. 10.1007/s10668-016-9760-4

[B27] Lang C. Popko J. Wirtz M. Hell R. Herschbach C. Kreuzwieser J. (2007). Sulphite oxidase as key enzyme for protecting plants against sulphur dioxide. *Plant Cell Environ.* 30 447–455. 10.1111/j.1365-3040.2006.01632.x 17324231

[B28] Leustek T. Martin M. N. Bick J. A. Davies J. P. (2000). Pathways and regulation of sulfur metabolism revealed through molecular and genetic studies. *Annu. Rev. Plant Physiol. Plant Mol. Biol.* 51 141–165. 10.1146/annurev.arplant.51.1.141 15012189

[B29] Leustek T. Saito K. (1999). Sulfate transport and assimilation in plants. *Plant Physiol.* 120 637–644. 10.1104/pp.120.3.637 10398698 PMC1539218

[B30] Livaka K. J. Schmittgen T. D. (2001). Analysis of relative gene expression data using real-time quantitative PCR and the 2^-ΔΔCt^ method. *Methods* 25 402–408. 10.1006/meth.2001.1262 11846609

[B31] Lopes M. S. Araus J. L. van Heerden P. D. Foyer C. H. (2011). Enhancing drought tolerance in C(4) crops. *J. Exp. Bot.* 62 3135–3153. 10.1093/jxb/err105 21511912

[B32] Lou L. Kang J. Pang H. Li Q. Du X. Wu W. (2017). Sulfur protects pakchoi (*Brassica chinensis* L.) seedlings against cadmium stress by regulating ascorbate-glutathione metabolism. *Int. J. Mol. Sci.* 18:E1628. 10.3390/ijms18081628 28933771 PMC5578019

[B33] Nagalakshmi N. Prasa M. N. (2001). Responses of glutathione cycle enzymes and glutathione metabolism to cooper stress in *Scenedesmus bijugatus*. *Plant Sci.* 160 291–299. 10.1016/S0168-9452(00)00392-7 11164601

[B34] Nahar K. Hasanuzzaman M. Alam M. M. Rahman A. Mahmud J. A. Suzuki T. (2017). Insights into spermine-induced combined high temperature and drought tolerance in mung bean: osmoregulation and roles of antioxidant and glyoxalase system. *Protoplasma* 254 445–460. 10.1007/s00709-016-0965-z 27032937

[B35] Pasternak M. Lim B. Wirtz M. Hell R. Cobbett C. S. Meyer A. J. (2008). Restricting glutathione biosynthesis to the cytosol is sufficient for normal plant development. *Plant J.* 53 999–1012. 10.1111/j.1365-313X.2007.03389.x 18088327

[B36] Randewig D. Hamisch D. Herschbach C. Eiblmeier M. Gehl C. Jurgeleit J. (2012). Sulfite oxidase controls sulfur metabolism under SO2 exposure in *Arabidopsis thaliana*. *Plant Cell Environ.* 35 100–115. 10.1111/j.1365-3040.2011.02420.x 21895698

[B37] Schrader N. Fisher K. Theis K. Mendel R. R. Schwarz G. Kisker C. (2003). The crystal structure of plant sulfite oxidase provides insights into sulfite oxidation in plants and animals. *Structure* 11 1251–1263. 10.1016/j.str.2003.09.001 14527393

[B38] Schroeder J. I. Kwak J. M. Allen G. J. (2001). Guard cell abscisic acid signaling and engineering drought hardiness in plants. *Nature* 410 327–330. 10.1038/35066500 11268200

[B39] Shinozaki K. Yamaguchi-Shinozaki K. (2007). Gene networks involved in drought stress response and tolerance. *J. Exp. Bot.* 58 221–227. 10.1093/jxb/erl164 17075077

[B40] Singh B. Bohra A. Mishra S. Joshi R. Pandey S. (2015). Embracing new-generation ‘omics’ tools to improve drought tolerance in cereal and food-legume crops. *Biol. Plant.* 59 413–428. 10.1007/s10535-015-0515-0

[B41] Singh M. Kushwaha B. K. Singh S. Kumar V. Singh V. P. Prasad S. M. (2017). Sulphur alters chromium (VI) toxicity in *Solanum melongena* seedlings: role of sulphur assimilation and sulphur-containing antioxidants. *Plant Physiol. Biochem.* 112 183–192. 10.1016/j.plaphy.2016.12.024 28088020

[B42] Su X. Wei F. Huo Y. Xia Z. (2017). Comparative physiological and molecular analyses of two contrasting flue-cured tobacco genotypes under progressive drought stress. *Front. Plant Sci.* 8:827. 10.3389/fpls.2017.00827 28567053 PMC5434153

[B43] Takahashi H. Kopriva S. Giordano M. Saito K. Hell R. (2011). Sulfur assimilation in photosynthetic organisms: molecular functions and regulations of transporters and assimilatory enzymes. *Annu. Rev. Plant Biol.* 62 157–184. 10.1146/annurev-arplant-042110-103921 21370978

[B44] Wang M. Jia Y. Xu Z. Xia Z. (2016). Impairment of sulfite reductase decreases oxidative stress tolerance in *Arabidopsis thaliana*. *Front. Plant Sci.* 7:1843. 10.3389/fpls.2016.01843 27994615 PMC5133253

[B45] Weckopp S. C. Kopriva S. (2014). Are changes in sulfate assimilation pathway needed for evolution of C4 photosynthesis? *Front. Plant Sci.* 5:773. 10.3389/fpls.2014.00773 25628630 PMC4292454

[B46] Xia Z. Su X. Liu J. Wang M. (2013). The RING-H2 finger gene 1 (*RHF1*) encodes an E3 ubiquitin ligase and participates in drought stress response in *Nicotiana tabacum*. *Genetica* 141 11–21. 10.1007/s10709-013-9702-0 23381133

[B47] Xia Z. Su X. Wu J. Wu K. Zhang H. (2012a). Molecular cloning and functional characterization of a putative sulfite oxidase (SO) ortholog from *Nicotiana benthamiana*. *Mol. Biol. Rep.* 39 2429–2437. 10.1007/s11033-011-0993-x 21667106

[B48] Xia Z. Sun K. Wang M. Wu K. Zhang H. (2012b). Overexpression of a maize sulfite oxidase gene in tobacco enhances tolerance to sulfite stress via sulfite oxidation and CAT-mediated H2O2 scavenging. *PLoS One* 7:e37383. 10.1371/journal.pone.0037383 22693572 PMC3365070

[B49] Yarmolinsky D. Brychkova G. Kurmanbayeva A. Bekturova A. Ventura Y. Khozin-Goldberg I. (2014). Impairment in sulfite reductase leads to early leaf senescence in tomato plants. *Plant Physiol.* 165 1505–1520. 10.1104/pp.114.241356 24987017 PMC4119034

[B50] Zhang X. Wong S. M. (2009). Hibiscus chlorotic ringspot virus upregulates plant sulfite oxidase transcripts and increases sulfate levels in kenaf (*Hibiscus cannabinus* L.). *J. Gen. Virol.* 90 3042–3050. 10.1099/vir.0.012112-0 19726610

