# Supplementary figures and images for: Overexpression of the Maize Sulfite Oxidase Increases Sulfate and GSH Levels and Enhances Drought Tolerance in Transgenic Tobacco
Source: Front Plant Sci. 2018 Mar 12;9:298. doi: 10.3389/fpls.2018.00298 (PMC5857591; doi:10.3389/fpls.2018.00298)

**Fig.S1**

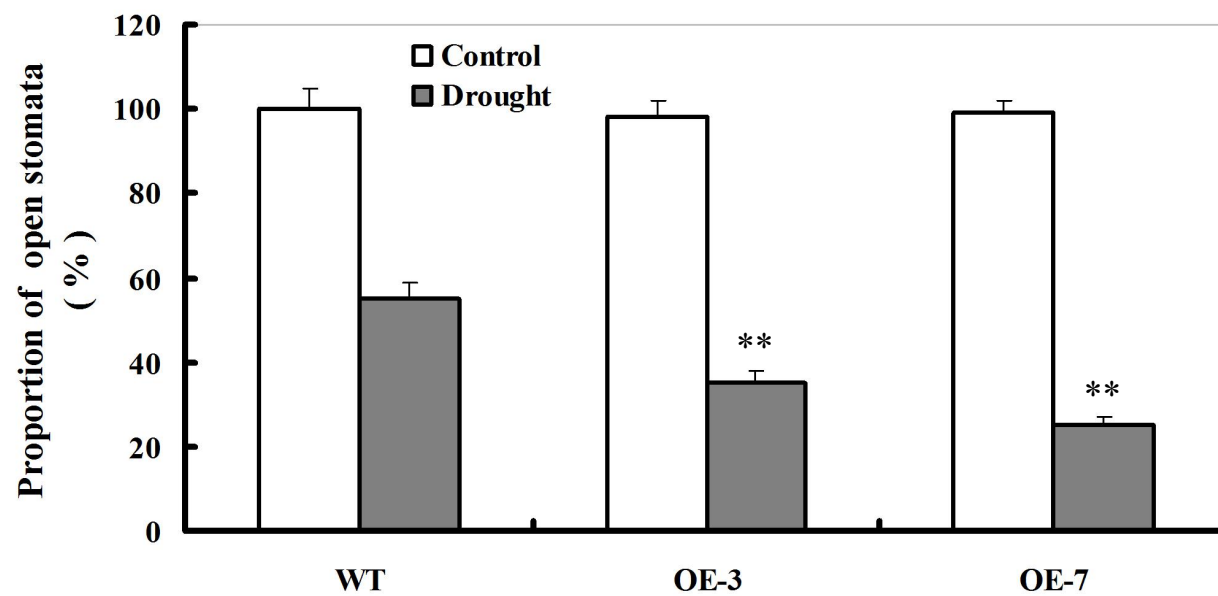

Supplement: FIGURE S1 — The proportion of open stomata in the wild-type (WT) and ZmSO- overexpressing tobacco lines under water stress. The proportion of open stomata was determined by the number of open stomata to that of stomata counted in epidermal peels from leaves of WT and both OE (OE-3 and OE-7) plants upon 9 days of control and drought stress. Bar indicates SE. ∗∗t-test, with P < 0.01. [file Image_1.PDF]
