# Supplementary material for: Overexpression of the Maize Sulfite Oxidase Increases Sulfate and GSH Levels and Enhances Drought Tolerance in Transgenic Tobacco
Source: Front Plant Sci. 2018 Mar 12;9:298. doi: 10.3389/fpls.2018.00298 (PMC5857591; doi:10.3389/fpls.2018.00298)
Supplement: TABLE S1 — qPCR primers used in this study. [file Table_1.PDF]

Supplementary data

Table S1 qPCR primers used in this study

Primer name	Primer sequence	Use
ZmSO-QF<br>ZmSO-QR	GTGGTCCTTATACGGCATCTATTC<br>CGTTGATACTGTCCAGCCATTTTA	qPCR for maize *SO* expression
NtSiR-QF<br>NtSiR-QR	5'-TGTAAAAGGAGCGATGAAGAAGG-3'<br>5'-AGGCCATTGCTGTTAGATTGAGT-3'	qPCR for tobacco *SiR* expression
NtAPR-QF<br>NtAPR-QR	5'-AGTTAGGACCCAATGGCTTTTCT-3'<br>5'-GTTCCAGGGGACTGATCTTTACG-3'	qPCR for tobacco *APR* expression
NtGSH1-QF<br>NtGSH1-QR	5'- ACCAGTAATGCCGAAGGGGAGAT-3'<br>5'-CAAGGCAAGACCAGCACGAAACT-3'	qPCR for tobacco *GSH1* expression
NtGSH2-QF<br>NtGSH2-QR	5'-CGGGGCTAAGTTGTCTTGTCAGT-3'<br>5'-TTGCGCTCTTCAGCTAGAATCAC-3'	qPCR for tobacco *GSH2* expression
NtActin2-QF<br>NtActin2-QR	5'-TGGCATCACACTTTCTACAA -3'<br>5'-CAACGGAATCTCTCAGCTCC-3'	qPCR for tobacco *Actin2* expression
ZmUbi-QF<br>ZmUbi-QR	5'-TAAGCTGCCGATGTGCCTGCGTCG-3'<br>5'-TGAAAGACAGAACATAATGAGCAC-3'	qPCR for maize *Ubi* expression
